# Changes in wet bulb globe temperature and risk to heat-related hazards in Bangladesh

**DOI:** 10.1038/s41598-024-61138-8

**Published:** 2024-05-06

**Authors:** A. S. M. Maksud Kamal, Abul Kashem Faruki Fahim, Shamsuddin Shahid

**Affiliations:** 1 Department of Disaster Science and Climate Resilience, University of Dhaka, Dhaka, 1000 Bangladesh; 2 Department of Water and Environmental Engineering, Faculty of Civil Engineering, Universiti Teknologi Malaysia, 81310 Johor Bahru, Malaysia

**Keywords:** Heat stress, Humidity, Solar radiation, Wind speed, Bangladesh, ERA5, Climate sciences, Environmental sciences, Health occupations, Risk factors

## Abstract

The rise in temperatures and changes in other meteorological variables have exposed millions of people to health risks in Bangladesh, a densely populated, hot, and humid country. To better assess the threats climate change poses to human health, the wet bulb globe temperature (WBGT) is an important indicator of human heat stress. This study utilized high-resolution reanalysis data from the fifth-generation European Centre for Medium-Range Weather Forecasts (ECMWF ERA5) to analyze the spatiotemporal changes in outdoor WBGT across Bangladesh from 1979 to 2021, employing Liljegren's model. The study revealed an increase in the annual average WBGT by 0.08–0.5 °C per decade throughout the country, with a more pronounced rise in the southeast and northeast regions. Additionally, the number of days with WBGT levels associated with high and extreme risks of heat-related illnesses has shown an upward trend. Specifically, during the monsoon period (June to September), there has been an increase of 2–4 days per decade, and during the pre-monsoon period (March to May), an increase of 1–3 days per decade from 1979 to 2021. Furthermore, the results indicated that the escalation in WBGT has led to a five-fold increase in affected areas and a three-fold increase in days of high and extreme heat stress during the monsoon season in recent years compared to the earlier period. Trend and relative importance analyses of various meteorological variables demonstrated that air temperature is the primary driver behind Bangladesh's rising WBGT and related health risks, followed by specific humidity, wind speed, and solar radiation.

## Introduction

The increasing temperatures have led to changes in atmospheric circulation patterns and geographic variations in meteorological factors, such as wind and humidity, which directly affect human comfort^1–3^. Therefore, relying solely on air temperature or simple thermal indices is insufficient to assess human thermal stress in a changing climate. Various indices that consider the combined effect of multiple meteorological variables related to human thermal stress have been proposed^4–6^. The Wet Bulb Globe Temperature (WBGT) was developed by the United States Army to estimate heat stress on the human body outdoors under direct sunlight^7^. It incorporates multiple meteorological variables, including air temperature, wind speed (WS), dew point temperature (Td), solar radiation (SR), surface pressure, sun angle, and surface albedo, to assess human thermal stress^8^. This enables WBGT to capture the influence of various atmospheric variables on thermal stress conditions, making it widely used for assessing thermal stress in changing climates^9,10^. The Health and Safety Executive (HSE) has recommended WBGT for evaluating thermal impacts on public health, labor productivity, ecology, and environmental sustainability^11^. Similarly, the International Organization for Standardization (ISO) has suggested WBGT as a screening tool for human thermal stress^12,13^.

Bangladesh, situated in Southern Asia, is a global hotspot for heat extremes^14^. The combination of increased temperature and humidity has led to a twofold rise in the heat index in many regions of South Asia, including Bangladesh, between 1979 and 2017^15,17^. Due to its proximity to the Bay of Bengal, Bangladesh experiences high humidity levels, making it particularly vulnerable to heat stress^18,19^. As Bangladesh is part of the Asian monsoon regime, the predominant southerly flows during the summer monsoon season increase the risk of heat stress by bringing warm and humid air from the Bay of Bengal to the country^20^. The associated dangers have also escalated with the intensification of climate change^21^. Bangladesh has witnessed a higher frequency of extreme heat in recent years than in previous years^22^.

The country's high population density and low adaptability have increased its vulnerability to heat stress^23^. Global warming of 1.5 °C would result in a 1% reduction in labor productivity in Asia and 2% in Southeast Asia^24^. In contrast, the International Labour Organization (ILO) estimated a 4.84% reduction in working hours in Bangladesh by 2030 due to increased heat stress. This significant impact is attributed to approximately 37.75% of the country's 160 million population being employed in agriculture, exposing them to prolonged heat stress^25^. Furthermore, nearly 61% of the population resides in rural areas with limited capacity to cope with heat stress^26^. The construction sector employs approximately 3.5 million people^27^, and 1.8 million individuals work as rickshaw pullers, a crucial mode of transport in the country^28^. The large population engaged in long-duration outdoor activities makes them highly susceptible to heat stress. A recent study indicated that a rise of 1 °C in temperature could lead to a loss of nearly 21 billion working hours annually in Bangladesh^29^. Additionally, the country experiences a significant number of temperature-related deaths. Burkart et al. (2014)^30^ estimated 22,840 temperature-related mortalities in Bangladesh each year, while Nissan et al.^31^ reported a 20% increase in heat-related deaths over the past decade. These findings underscore the substantial economic impact of heat stress in Bangladesh if adequate adaptation measures are not implemented.

The ongoing trend of increasing temperature and humidity will continue to exacerbate thermal stress conditions worldwide. Battisti and Naylor^32^ projected a more pronounced increase in the tropics and subtropics. The Intergovernmental Panel on Climate Change^33^ also predicted the prevalence of heatwaves in South Asia, including Bangladesh, even if the temperature rise is limited to below 1.5 °C. However, research on WBGT and heat stress changes in Bangladesh is limited. Previous studies in Bangladesh have mainly focused on severe temperature variability^34^, heat index (HI) trends^35^, and the consequences of heat extremes^30^. Similarly, studies on WBGT in neighboring regions are scarce. Monteiro and Caballero^36^ characterized extreme WBGT events in Southern Pakistan and their association with humid marine airflow from the Arabian Sea. Im et al.^37^ used global climate models to project WBGT in South Asia and highlighted the potential impact on densely populated regions such as the Indus and Ganges basins, including parts of Bangladesh. Saeed et al.^15^ identified many areas in South Asia with WBGT exceeding 32 °C, representing the upper limits of human survivability. Jacobs et al.^38^ partially assessed WBGT in three major South Asian cities and found significantly high heat indices, particularly during the summer season in Dhaka, Bangladesh.

The existing research on WBGT in Bangladesh has predominantly utilized constrained observed data, leading to a notable absence of thorough spatiotemporal trend evaluations for WBGT. The mounting temperatures have amplified processes like vaporization and alterations in humidity and other meteorological parameters. This climate shift has significantly reduced wind speed (WS), accompanied by increased cloud cover in the country^39–41^. The wide-ranging fluctuations in these meteorological factors across different parts of Bangladesh have showcased marked diversity, contributing to considerable spatial discrepancies in heat stress levels within the nation. Consequently, there is a substantial research gap concerning a comprehensive understanding of the intricate interactions between WBGT and diverse meteorological variables across different regions in Bangladesh.

This study utilized high-resolution gridded reanalysis climate data to assess the spatiotemporal changes in WBGT. This research represents the first attempt to conduct trend analysis on the spatiotemporal variations of WBGT in Bangladesh, incorporating multiple climatic variables such as the U and V components of wind, 2 m temperature (Ta), and dew point temperature (Td), surface net solar radiation (SR), surface net thermal radiation, surface pressure, total sky direct solar radiation, and surface downward solar radiation. The findings of this study can be instrumental in preparing for adaptation strategies and mitigating the impacts of climate change on disease and mortality. This is especially crucial, considering many economically vulnerable individuals engaged in construction and agriculture are exposed to excessive heat in the country^42^.

## Study area

Bangladesh, situated along the Bay of Bengal, spans between the latitudes of 20°34'N and 26°38'N and the longitudes of 88°01'E and 92°41'E, as shown in Supplementary Figure S1^43^. Positioned on both sides of the Tropic of Cancer, Bangladesh features a humid tropical/subtropical climate characterized by warm and pleasant conditions, significant seasonal variations in rainfall patterns, and relatively high humidity levels^44^. According to Beck et al.^45^ and Fahim et al.^46^, the country's land is divided into three distinct zones based on prevailing climate conditions. Most of the nation experiences a tropical climate, except for certain areas in the northwest with a dry winter and hot summer climate. Bangladesh typically receives rainfall ranging between 1600 to 4400 mm. In most parts of the country, the average annual relative humidity level exceeds 75%. The annual mean minimum and maximum temperatures are recorded at 21.4°C and 29.9°C, respectively. The country faces hot extremes during the hot and humid pre-monsoon summer (March to May) and the humid monsoon summer (June to September). Crop cultivation and harvesting predominantly occur during Bangladesh's pre-monsoon and monsoon seasons^47^. As a result, millions of individuals engaged in these agricultural activities are particularly susceptible to heat stress during these two seasons.

## Research methodology and materials

### Datasets

ERA5 is the fifth generation of reanalysis of the global climate and weather conducted by the European Centre for Medium-Range Weather Forecasts (ECMWF). With a horizontal resolution of 0.25° and a temporal resolution of 1 h, ERA5 is created using the most recent iteration of the Integrated Forecasting System. These data sets are accessible for various periods on a global and regional basis (https://cds.climate.copernicus.eu//cdsapp#!/dataset/reanalysis-era5-singlelevels?tab=overview). In this study, hourly averaged reanalysis datasets for the period 1979 to the present were used. The ERA5 variables used in the present study are given in Table 1.

**Table 1:** ERA5 hourly datasets and their details used in this study.

No	Parameter	Symbol	Unit
1	Wind (u component) at 10 m	u_10_	m/s
2	Wind (v component) at 10 m	v_10_	m/s
3	Dew point temperature at 2 m	d_2m_	K
4	Specific humidity	SH	kg/kg
5	Air temperature at 2 m	t_2m_	K
6	Surface Net Solar Radiation (SR)	s_sr_	Jm^−2^
7	Surface Net Thermal Radiation	s_tr_	Jm^−2^
8	Surface Pressure	s_p_	Pa
9	Total Sky Direct SR	*f*_dir_	Jm^−2^
10	Surface SR Downwards	ssrd	Jm^−2^

Several studies have demonstrated the outstanding performance of ERA5 data in replicating observed climate variables^48–51^. Islam and Cartwright^50^ showed ERA5's ability to measure rainfall and temperature accurately in Bangladesh. Zhai et al.^48^ demonstrated its capability to monitor South Asian drought. Kamruzzaman et al.^49^ used ERA5 as the reference data for their climate change projections. In a recent study, Yildiz et al.^51^ thoroughly evaluated the reliability of ERA5 data in Bangladesh, using observations from 29 meteorological stations. They confirmed ERA5's accuracy in replicating many meteorological variables' seasonal and temporal patterns.

ArcGIS Desktop 10.8 and RStudio software (built upon R version 4.2.0) are the main tools used for processing and analyzing data^52,53^. All the maps in this study have been prepared using the “Terra” package^54^ in R other than the Supplementary Figure S1. Supplementary Figure S4 was prepared using ArcGIS.

### Methods

The main steps followed to attain the objectives are: (a) collecting and pre-processing ERA5 hourly datasets, (b) calculating WBGT of Bangladesh from 1979 to 2021 based on the method by Liljegren et al.^54^ using the processed ERA5 data, (c) analyzing the trends of average and maximum annual, pre-monsoon and monsoon WBGT, and (d) analyzing the average and maximum annual, pre-monsoon and monsoon trends of the drivers of WBGT, i.e. relative humidity (RH), *Ta*, SR, and WS.

The Liljegren method of WBGT calculation is the most reliable and accurate alternative to direct measurement^11,55^. Various studies have demonstrated its high accuracy^56,57^. Therefore, the Liljegren method is considered a benchmark for WBGT estimation and has been widely recommended for calculating outdoor WBGT in climate change assessments. Kong and Huber^11^ recognized the Liljegren method as a well-validated, explicit physical model for WBGT estimation, performing well with ERA5 reanalysis data. The process of calculating WBGT, according to Liljegren et al.^54^ using the ERA5 datasets, is discussed in the following section.

#### Outdoor wet bulb globe temperatures (WBGT)

*Outdoor wet bulb globe temperatures* (*WBGT*): WBGT was calculated using standard meteorological data at each ERA5 grid. The weighted sum of natural wet bulb temperature (T_nwb_), dry bulb/ambient temperature (T_amb_), and globe temperature (T_g_) is used to compute outdoor WBGT^54^:

1 $$WBGT=0.7 {T}_{nwb} \times 0.1{T}_{amb} \times 0.2{T}_{g}$$

T_nwb_ and T_g_ must be estimated to derive WBGT using standard readings. Creating separate models for the natural wet bulb and globe temperatures is important to represent the WBGT effectively. Different atmospheric, psychrometric, and land surface variables are used to estimate different components of WBGT, which is pictorially presented in Supplementary Figure S2.

*Natural wet bulb globe temperature* (*T*_*nwb*_): Wet wick temperature sensors measure *T*_*nwb*_ and the aspirated or psychrometric wet bulb temperature (*T*_*pwb*_). The sensor is often encased in an inhalation radiation shield to separate the evaporative cooling process from other variables during the Tpwb measurement. Because the wick is left exposed during a *T*_*nwb*_ test, factors such as solar and thermal radiative heat transfer and variable convective heat transmission can alter the measured temperature. Therefore, the *T*_*nwb*_ represents the effect of these processes on the human body's ability to cool itself through perspiration. For either the wick's *T*_*pwb*_ or *T*_*nwb*_ temperature, the mass balance equation^58^ is:

2 $$\omega (1 - {x}_{w}) = {k}_{x}A ({x}_{w}- {x}_{a})$$

where *A* is the wick's surface area, which is the Cylinder's surface area having a length of L and diameter of D, such that *A* = *πDL*, and w is the molar water vapor flux from the wick to the air, ${x}_{a}$ is the water vapor's ambient mole fraction, ${x}_{w}$ is the water vapor's mole fraction in the air at the surface of the wick, and ${k}_{x}$ refers to the convective mass transfer coefficient. The water vapor taken in by the air is represented by the word to the right of the equal sign, while the term represents the water vapor lost by the wick due to evaporation to the left of the equal sign. Ideal gas law allows for the following expression of the mole fraction:

3 $$x=\frac{{n}_{vapor}}{{n}_{total}}=\frac{e}{P}$$

where *e* indicates water vapor's partial pressure, *n* represents mole number, and *P* is the barometric pressure. The RH (reported as a proportion of saturation) and the saturation vapor pressure *e*_*sat*_ at *T*_*amb*_ determined employed Buck^59^ method is used to determine the water vapor's partial pressure. Putting Eq. (3) back into Eq. (2) and figuring out *ω* produces:

4 $$\omega ={k}_{x}A\left(\frac{{e}_{w}-{e}_{a}}{P-{e}_{w}}\right)$$

The wick's energy balance can be expressed as^58^:

5 $$\omega {M}_{{H}_{2}O}\Delta H=hA\left({T}_{amb}-{T}_{w}\right)+\Delta {F}_{net}$$

where $\Delta {F}_{net}$ is the flux of net radiant heat flow to the wick from the environment ($\Delta {F}_{net}$ = 0 indicates that the T_pwb_), ${T}_{w}$ is the temperature of the wick, ${T}_{amb}$ is the ambient temperature, *h* represents the convective heat transfer coefficient, $\Delta H$ is the vaporization heat, while ${M}_{{H}_{2}O}$ stands for the molecular weight of water vapor. The energy that the wick loses through evaporation is represented by the word to the left of the equal sign, while the right-side term reflects the wick's energy gains through convection and radiation. Equation (2) and (5) may be combined to get ${T}_{w}$^58^ :

6 $${T}_{w}={T}_{amb}-\frac{{k}_{x}{M}_{{H}_{2}O}\Delta H}{h}\left(\frac{{e}_{w}-{e}_{a}}{P-{e}_{w}}\right)+\frac{\Delta {F}_{net}}{Ah}$$

The equivalence of heat transfer and mass transfer can be used to determine the ratio *k*_*x*_*/h*:

7 $${j}_{H}={j}_{D}$$

where the Chilton-Colburn* j*-factors^60^ represent heat and mass transfer, respectively, are ${j}_{H}$ = N_u_ R_e_^−1^ Pr^a−1^ and ${j}_{D}$ = S_h_ R^e−1^ S_c_^a−1^. The Nusselt number (N_u_equalsto hD/k, where k and h represent the fluid's thermal conductivity and the Cylinder's convective heat transfer coefficient, respectively. The Reynolds number, Re = *ρVD/µ*, where D is the Cylinder's diameter, and *V*, *ρ,* and* µ* are, respectively, the velocity, density, and viscosity of the fluid. The formula for the Prandtl number, P_r_ = *c*_*p*_*µ/k*, where *cp* represents specific heat under constant pressure. The Sherwood number, or S_h_, is the Nusselt number's mass transfer equivalent: S_h_ = *k*_*x*_*MD/*(*D*), where *kx* represents convective mass transfer, *M* represents fluid's average molecular weight (dry air), and D represents diffusivity (of water coefficient vapor in the air). The mass transfer equivalent of the Prandtl number is the Schmidt number, S_c_ = *µ*/(*ρD*). From Eq. (7), *k*_*x*_*/h*, may be solved to provide:

8 $$\frac{{k}_{x}}{h}=\frac{1}{{c}_{p}{M}_{Air}}{\left(\frac{{P}_{r}}{{S}_{c}}\right)}^{a}$$

and replaced again into Eq. (6)

9 $${T}_{w}={T}_{amb}-\frac{{M}_{{H}_{2}O}\Delta H}{{M}_{Air}{c}_{p}}\left(\frac{{e}_{w}-{e}_{a}}{P-{e}_{w}}\right){\left(\frac{{P}_{r}}{{S}_{c}}\right)}^{a}+\frac{\Delta {F}_{net}}{Ah}$$

The dimensionless, empirical correlation provided by Bedingfield and Drew^61^ for Cylinder's heat or mass transfer is used to calculate the convective heat transfer coefficient ($\Delta {F}_{net}/Ah)$ in Eq. (9):

10 $${N}_{u}{{R}_{e}}^{-1}{{P}_{r}}^{a-1}={{bR}_{e}}^{-c}$$

where c = 0.4, b = 0.281, and a = 0.56, and. $h=k/D b {{R}_{e}}^{1-c}{{P}_{r}}^{1-a}$ is the result obtained from the convective heat transfer coefficient. According to Eq. (9), the association between the convective heat transfer coefficient and the Re in $\Delta {F}_{net}/Ah$ demonstrates how the *T*_*nwb*_ is dependent on WS. Since the term $\Delta {F}_{net}/Ah$ vanishes when $\Delta {F}_{net}$ = 0, Eq. (9) is reduced to the T_pwb_. The wick's net radiative gain from the surroundings is^58^:

11 $$\Delta {F}_{net}=A\sigma {\varepsilon }_{w}\frac{1}{2}\left({\varepsilon }_{a}{{T}_{amb}}^{4}+{\varepsilon }_{sfc}{{T}_{sfc}}^{4}\right)-A\sigma {\varepsilon }_{w}{{T}_{w}}^{4}+{A}_{1}\left(1-{a}_{w}\right)\left(1-{f}_{dir}\right)S+\frac{{A}_{2}\left(1-{a}_{w}\right){f}_{dir}S}{{\text{cos}}\left(\theta \right)}+A(1-{a}_{w}){\alpha }_{sfc}S$$

The right side term represents the wick's absorbance of the thermal radiation emitted from the ground surface, where *σ* represents the Stefan-Boltzmann constant, ${\varepsilon }_{w}$*,* ${\varepsilon }_{sfc}$, and ${\varepsilon }_{a}$ represent sick's, surface, and atmospheric emissivity, respectively, and ${T}_{sfc}$ represents surface temperature. The second term describes the heat radiation that the wick emits. The third and fourth terms represent the diffuse and direct solar irradiance absorbed by the wick, respectively, where ${A}_{1}$ and ${A}_{2}$ represent the wick's areas engaged in the transfer of radiation; ${a}_{w}$ indicates the wick's albedo; ${f}_{dir}$ indicates the portion of the total horizontal solar irradiance S that is due to the sun's direct beam; (1 − ${f}_{dir}$) is the portion due to the diffusion of solar irradiance, *θ* denotes the solar zenith angle, and ${\alpha }_{sfc}$ is the surface albedo.

Globe temperature (*T*_*g*_): The ${T}_{g}$ is monitored using a hollow black-coated metal with a thermometer inside. It acts as a stand-in for how these processes affect the human body due to its complete exposure to the environment's convective and radiative processes. The energy balance equation for the globe is^54^:

12 $$A{\varepsilon }_{g}\sigma {{T}_{g}}^{4}+Ah\left({T}_{g}-{T}_{amb}\right)=\frac{A}{2}{\varepsilon }_{g}\sigma \left({\varepsilon }_{a}{{T}_{amb}}^{4}+{\varepsilon }_{sfc}{{T}_{sfc}}^{4}\right)+\frac{A}{2}\left(1-{\alpha }_{g}\right)\left(1-{f}_{dir}\right)S+\frac{A}{4}\frac{\left(1-{\alpha }_{g}\right){f}_{dir}S}{{\text{cos}}\left(\theta \right)}+\frac{A}{2}\left(1-{\alpha }_{g}\right){\alpha }_{sfc}S$$

At the equal sign's left, the two components stand for the energy the earth loses through convection and thermal radiation, respectively. *A* represents the area of the sphere; *ε*_*g*_ represents sphere's emissivity. An empirical equation is employed to get the convective heat transfer coefficient:

13 $${N}_{u}{{=2.0+0.6R}_{e}}^{1/2}{{P}_{r}}^{1/3};h=k/D{N}_{u}$$

The energy acquired by the globe due to atmospheric and surface thermal radiation is represented by the first term in Eq. (12) to the right of the equal sign. The other two terms on the right side represent the energy acquired by the sphere from diffuse and direct solar irradiation. The absorbed SR by the sphere that is reflected from the surface represents the last right term in Eq. (12). After rearranging^54^:

14 $${{T}_{g}}^{4}=\frac{1}{2}\left(1+{\varepsilon }_{a}\right){{T}_{amb}}^{4}-\frac{h}{{\varepsilon }_{g}\sigma }\left({T}_{g}-{T}_{a}\right)+\frac{S}{2{\varepsilon }_{g}\sigma }\left(1-{\alpha }_{g}\right)\left[1+\left(\frac{1}{2{\text{cos}}\left(\theta \right)}-1\right){f}_{dir}+{\alpha }_{sfc}\right]$$

*Estimation of WS*: Mean WS is calculated from the wind components of ERA5 datasets through the following formula.

15 $${w}_{10}=\sqrt{{{(u}_{10})}^{2}+{{(v}_{10})}^{2}}$$

Since WS components of ERA5 datasets are calculated at 10-m height (${w}_{10}$), it is required to convert at 2 m height (${w}_{2}$) to calculate WBGT. A logarithmic WS profile was employed for this purpose^62^.

16 $${w}_{2}={w}_{10 }\times \frac{4.87}{\mathrm{ln }( 67 . 8 \times 10 - 5 . 42 )}$$

*Calculation of solar zenith angle*: The angle formed between sunbeams and earth's perpendicular or solar zenith angle varies with times and locations. It can be estimated using Pielke^63^,

17 $${\text{cos}}\theta ={\text{sin}}\varphi {\text{sin}}{\delta }_{sun}+{\text{cos}}\varphi {\text{cos}}{\delta }_{sun}{\text{cos}}{h}_{r}$$

where $\theta$ is solar zenith angle, ${\delta }_{sun}$ is the sun declination (ranges between − 23.5° on December 22 and + 23.5º on June 21), $\varphi$ is latitude, and ${h}_{r}$ is the hour angle (0º ≡ noon).

#### Trend analysis

Trend identification and/or quantification can be made using parametric, nonparametric, and mixed methods^64^. Nonparametric methods are widely used in climatic and hydrological research due to their resiliency to missing data, linked data, serial dependency, non-linearity, non-normality, and seasonality^64^. The Sen slope estimator^65^ and the nonparametric Mann–Kendall (M–K) test were both used in this study. The MK test is used because data homogeneity does not affect its statistics^66^. However, the presence of significant autocorrelation may affect its outcomes^16^. Therefore, this study used a modified version of the M–K test^67^, which uses the Hurst coefficient to characterize self-similar decay in time-series autocorrelation to remove its effect on test significance. This study employed modified M–K test to evaluate the significance of the shift, whereas the Sen slope estimator to measure changes per unit of time.

#### WBGT heat stress categories

The present study uses the WBGT heat stress categories of the United States Department of the Army, as presented in Table 2. Various organizations have established categories for WBGT to determine the maximum occupational heat exposure limit, assess worker's performance, identify heat-related health issues, and recommend appropriate clothing^68–72^. The current study employed the WBGT heat categories from the United States Department of the Army classification system to evaluate heat stress^7^. This classification is widely used to define heat illness risk levels^73,74^. The University of Georgia also adopted these categories to be used in collegiate athletics as a benchmark for how demanding practices may be (https://en.wikipedia.org/wiki/Wet-bulb_globe_temperature).

**Table 2:** Wet bulb globe temperature categories and corresponding heat stress levels.

Category	WBGT (°C)	Event conditions
1	25.6 − 27.8	Good conditions or no stress
2	27.8 − 29.4	Less than ideal conditions or mild risk
3	29.4 − 31.1	Moderate risk of heat-related illness
4	31.1 − 32.2	High risk of heat-related illness
5	> 32.2	Extreme conditions

## Results

### Geographical variability of WBGT in Bangladesh

**Figure 1:**
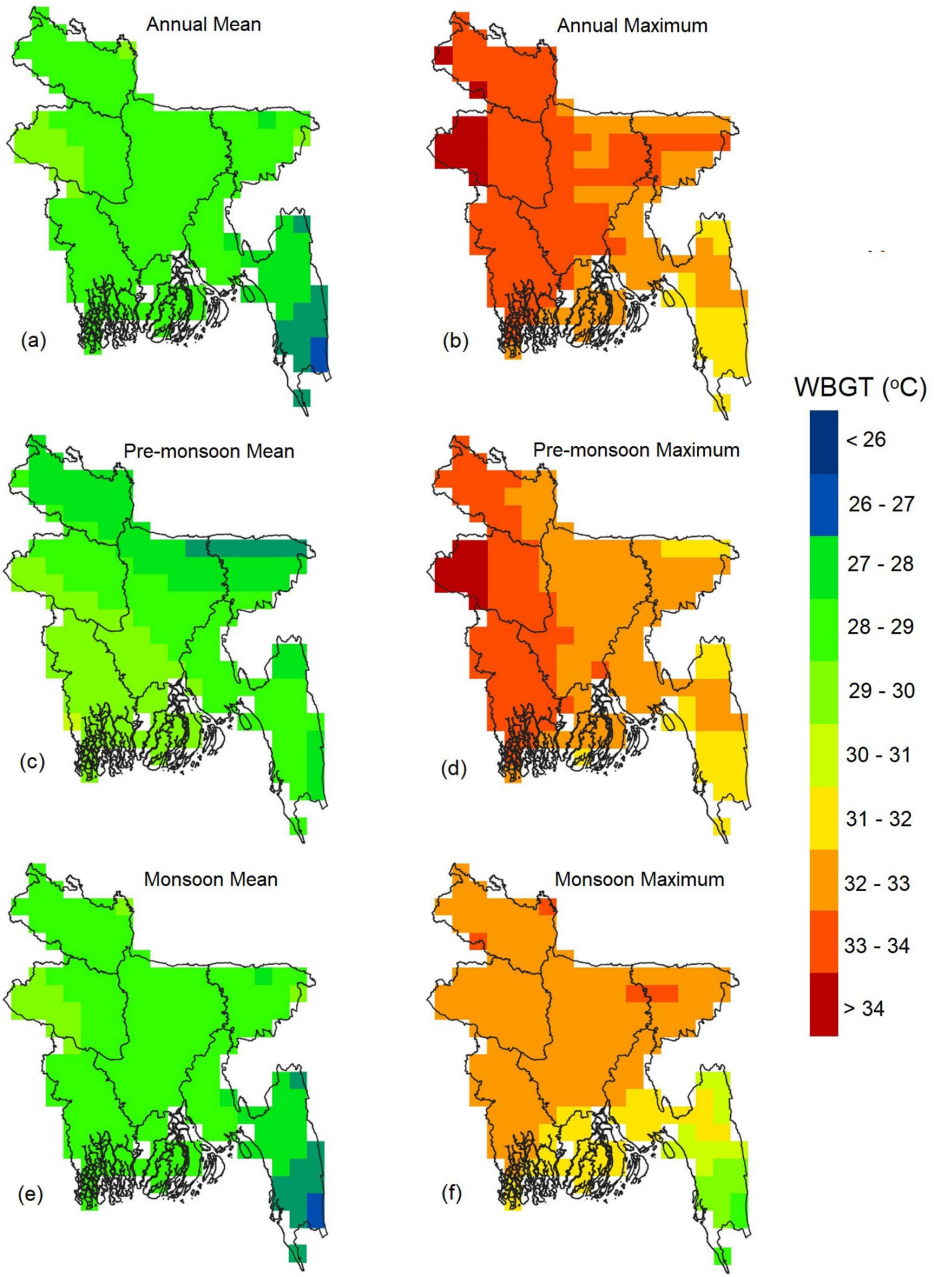
Geographical distribution wet bulb globe temperature (**a**) annual average; (**b**) annual maximum; (**c**) pre-monsoon average; (**d**) pre-monsoon maximum; (**e**) monsoon average; (**f**) monsoon maximum.

Figure 1 illustrates the geographical variability of Bangladesh's annual and seasonal daily average and maximum WBGT. The map was generated using WBGT calculations at each ERA5 grid point with a spatial resolution of 0.25° × 0.25°. The annual WBGT in the country ranges from 25 °C in the far southeast to 31 °C in the northwest (Fig. 1a). However, most of the country experiences a range of 29–30 °C. The geographical pattern of WBGT during the monsoon season is similar to the annual pattern but with a larger area in the range of 30–31 °C, covering the entire southwest and significant portions of the central-west and northwest regions (Fig. 1e). In the pre-monsoon season, areas with an average WBGT below 26 °C are more extensive in the northwest (Fig. 1c). Generally, the average WBGT, annual and seasonal, is relatively lower in the southwest and northwest highlands compared to the rest of the plains. The average temperature in Bangladesh typically decreases from the southeast to the northwest, except for the pre-monsoon WBGT, which follows a similar pattern to the average temperature.

Figure 1b depicts the geographical variability of Bangladesh's yearly average maximum WBGT. It shows that the country experiences an average maximum WBGT above 31 °C, reaching over 33 °C in the western half and peaking at 34.6 °C in the northwest. These results indicate that the entire country, except for the southwest hilly region, experiences extreme WBGT at least once a year. The maximum WBGT during the pre-monsoon season follows a pattern similar to the annual maximum but with slightly lower values. The northeast region also experiences lower WBGT (31–32 °C) during the pre-monsoon season. In the monsoon season (Fig. 1f), the maximum WBGT ranges between 32–33 °C in most parts of the country, except for the southeast, where it is less than 30 °C. The WBGT is also lower in the southern coastal region. The smaller coverage area of high WBGT temperatures in the monsoon and pre-monsoon seasons compared to the annual pattern suggests that higher maximum WBGT temperatures also occur in the post-monsoon season, particularly in the central region of Bangladesh.

### Trends in WBGT

**Figure 2:**
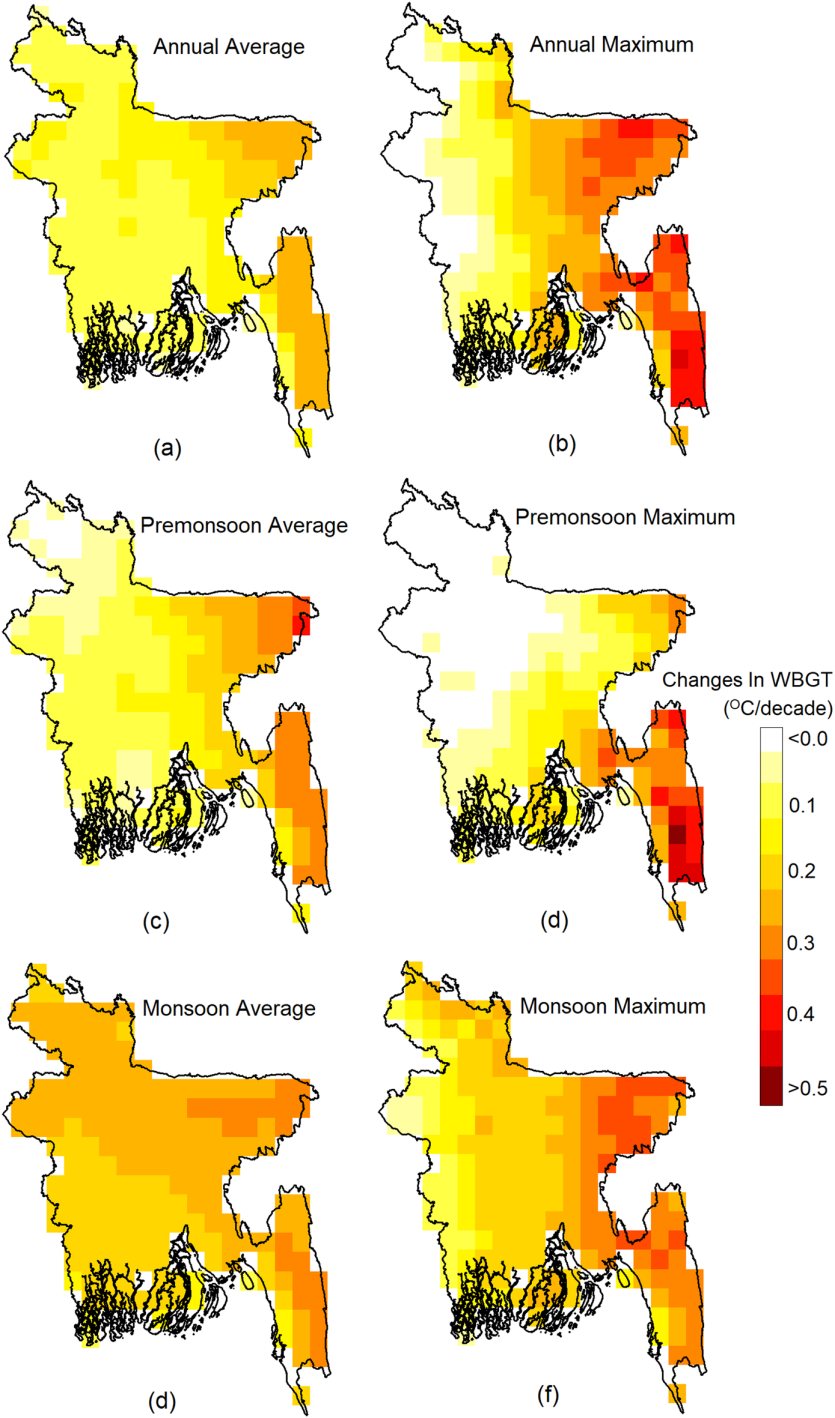
Changes in (**a**) annual average; (**b**) annual maximum; (**c**) pre-monsoon average; (**d**) pre-monsoon maximum; (**e**) monsoon average; (**f**) monsoon maximum WBGT for the period 1979–2021. All the changes shown in the maps are significant at a 95% confidence interval.

Figure 2 illustrates the geographical variability of changes in Bangladesh's annual and seasonal mean and maximum WBGT. The colour ramp represents the change in °C/decade, estimated using Sen's slope. Based on the Mann–Kendall test, all changes were significant at a significance level of *p* = 0.05. Therefore, significant trends are not marked with specific symbols in the figure. The results indicate an increase in annual and seasonal WBGT, with higher rates of increase observed in the southeast and northeast regions where WBGT is relatively lower and relatively lower increases in the west where WBGT is higher. This suggests a gradual homogenization of WBGT across the country. The highest increase was estimated for the annual average and pre-monsoon maximum WBGT in the southeast, with a rate of more than 0.5 °C per decade. Among the different time scales, the highest increases were observed for the annual average WBGT, while the lowest increases were observed for the monsoon average WBGT. In contrast, the highest increase in maximum WBGT was observed during the monsoon season, and the least increase was observed for the annual maximum WBGT. These results indicate that increases in WBGT during other seasons contribute more to the overall increase in annual average WBGT than increases during the monsoon season.

In the subsequent sub-sections, the maps presented in this section provide an overview of annual and seasonal WBGT changes. However, it is important to understand how these changes may impact human heat stress in Bangladesh. Therefore, the trends in different categories of WBGT, along with their spatial distribution across the country, are analyzed in the following sections.

### Trends in different categories of WBGT

**Figure 3:**
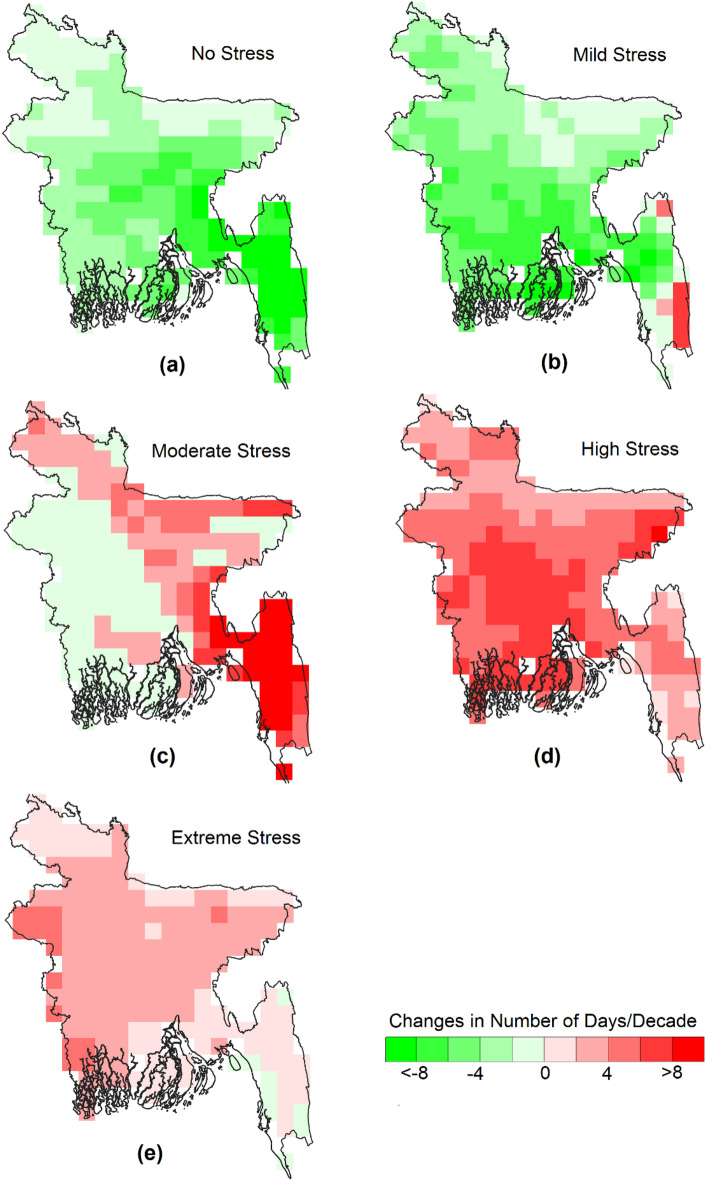
The spatial distribution of the trends in the number of days with different categories of annual WBGT related to heat stress. All the changes shown in the maps are significant at a 95% confidence interval.

Figure 3 presents the trends in the number of days with different categories of annual WBGT temperature related to heat stress. The analysis used daily WBGT data for the years 1979–2021 to estimate the trends. The results show that increased annual WBGT has led to a decline in the number of days with no stress or mild stress and an increase in the number of days with moderate to extreme heat stress. The highest increase was observed for high heat stress days, ranging from 2 to 8 days per decade for the entire country. There was also an increase of more than eight moderate stress days in the southeast hilly region, where WBGT is generally lower. The number of annual extreme heat stress days increased in the west, where WBGT is higher. Overall, the results indicate a growing risk of heat stress, with regions experiencing lower WBGT facing more moderate stress days and regions with higher WBGT facing higher extreme heat stress days.

**Figure 4:**
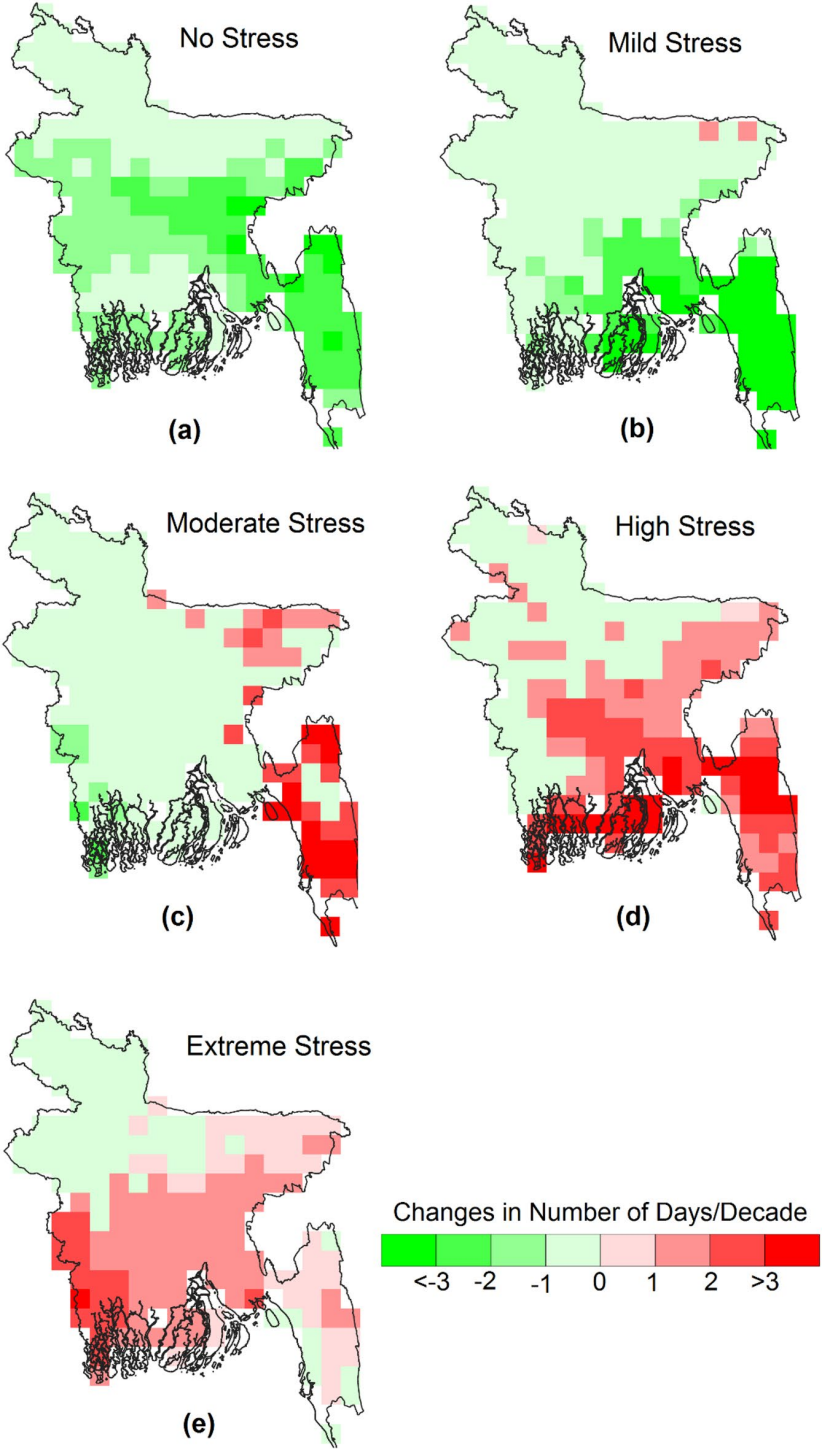
The spatial distribution of the trends in the number of days with different categories of pre-monsoon WBGT related to heat stress. All the changes shown in the maps are significant at a 95% confidence interval.

**Figure 5:**
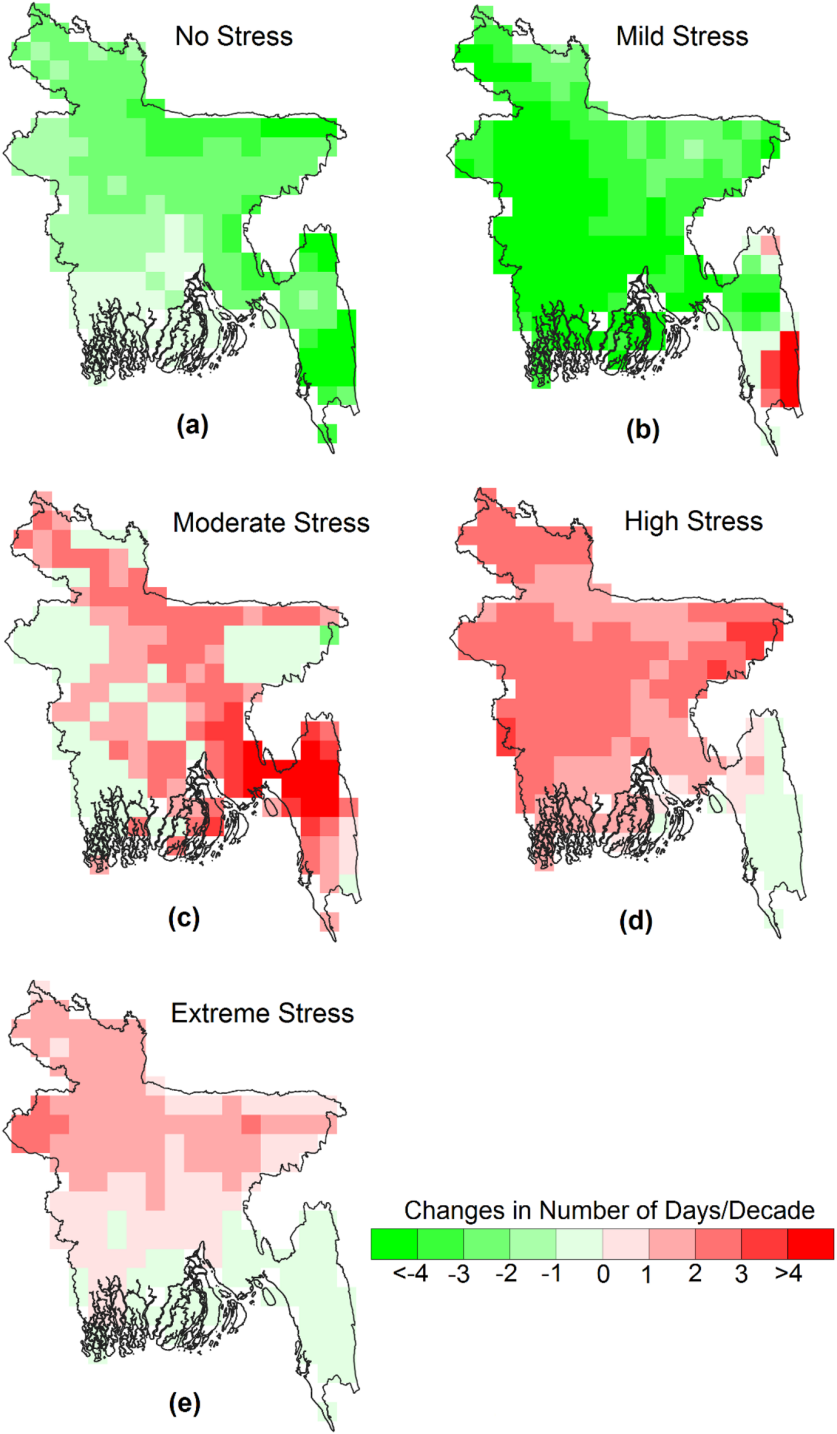
The spatial distribution of the trends in the number of days with different categories of monsoon WBGT related to heat stress. All the changes shown in the maps are significant at a 95% confidence interval.

Figures 4 and 5 depict the trends in pre-monsoon and monsoon days, respectively, categorized by heat stress levels. The results show similar trends in both seasons, with an overall increase in moderate heat stress days in the east, high heat stress days in most parts of the country, particularly in the central region, and extreme heat stress days in the west. The increase in monsoon high heat stress and extreme stress days is greater (2 to 4 days) than pre-monsoon heat stress days (1 to 3 days). There is a difference in the trends between pre-monsoon and monsoon for high and extreme heat-stress days. Monsoon high and extreme heat stress days decrease in the southeast, while pre-monsoon high heat stress days increase in the southeast. Pre-monsoon extreme stress days increase in the south but decrease in the north. The pattern is the opposite for monsoon extreme heat stress days. Although the number of no-stress and mild-stress days decreases almost throughout the country, the decrease is smaller in the pre-monsoon season compared to the monsoon. Mild heat stress days decrease by more than three days in most parts of the country, which is attributed to a higher increase in monsoon WBGT than pre-monsoon WBGT.

### Trends in spatial coverage and temporal extent in WBGT

**Figure 6:**
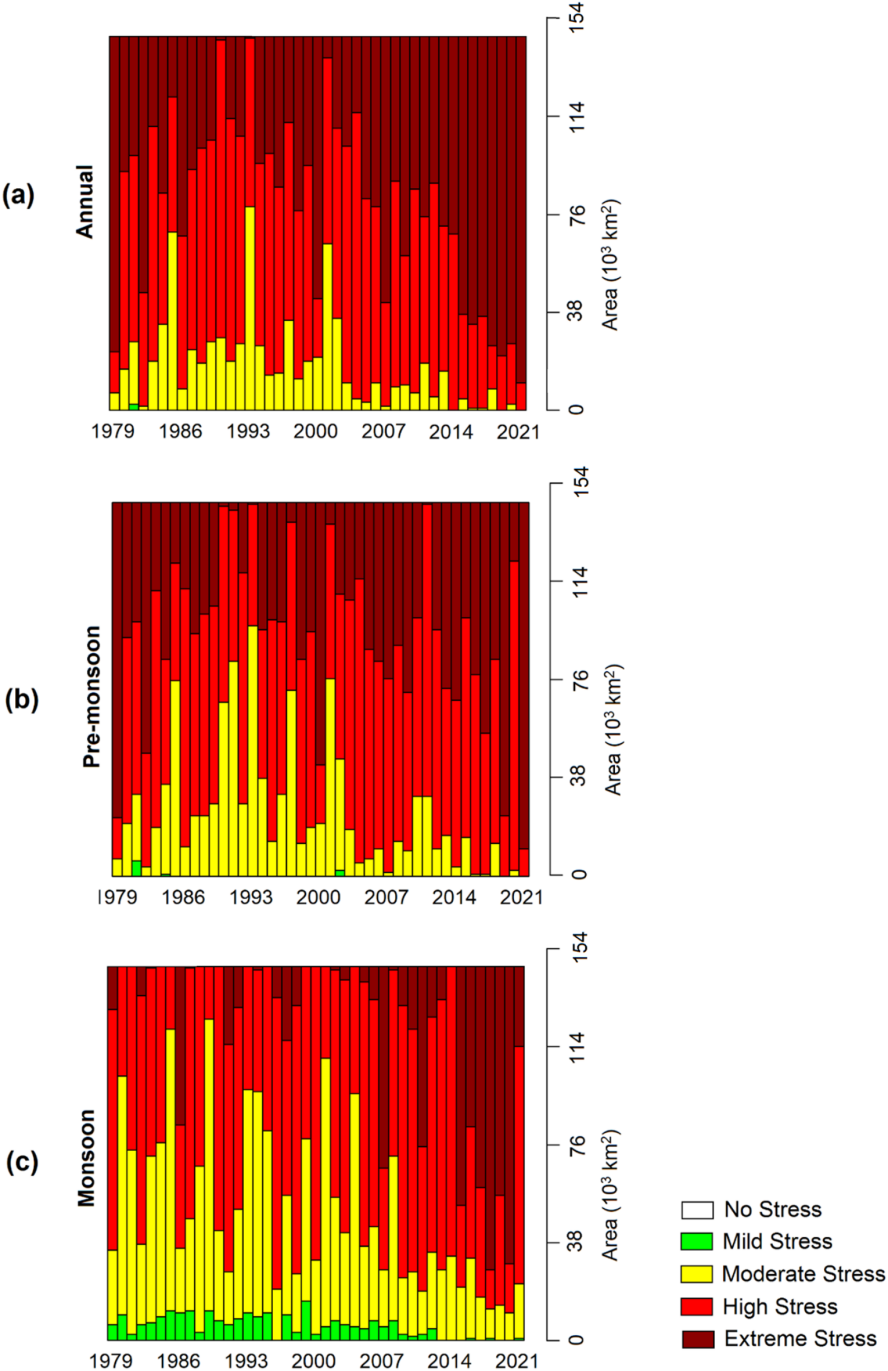
The area affected by different categories of maximum heat stress during (**a**) annual, (**b**) pre-monsoon, and (**b**) monsoon from 1979 to 2021 in Bangladesh.

To illustrate the changes in the affected areas by different categories of heat stress, Fig. 6 presents the estimated area affected by heat stress for each year during the study period. The graph shows different colors representing the affected areas by different categories of heat stress. The bars in the graph represent the maximum affected area in a year, as the areal average maximum WBGT data was used. The results reveal a gradual increase in the affected area by extreme heat stress in the country. Before 2001, extreme heat stress events that affected more than 50% of the country (approximately 72 thousand km^2^) were rare. Such events occurred in 1979, 1982, and 2000 before 2001. However, they have become more frequent since 2014. In contrast, the area affected by moderate heat stress gradually decreased. These findings indicate that the increased annual maximum WBGT has led to an expansion of the affected area by extreme heat stress in the country.

Similar trends of increased area coverage of extreme heat stress and decreased coverage of moderate stress are observed in both pre-monsoon and monsoon seasons. The largest increase in the affected area is seen during the monsoon for extreme heat stress. Previously, such events during the monsoon were less frequent and covered smaller areas, but they have now become common. The areal extent of extreme heat stress events has also significantly increased in the last decade, covering over 50% of the country's land. The coverage area of moderate stress during the monsoon has shown a drastic decline, and the coverage area of mild heat stress has become negligible in recent years. These results indicate a higher impact on the population and agriculture from extreme and high heat stress annually and seasonally.

**Figure 7:**
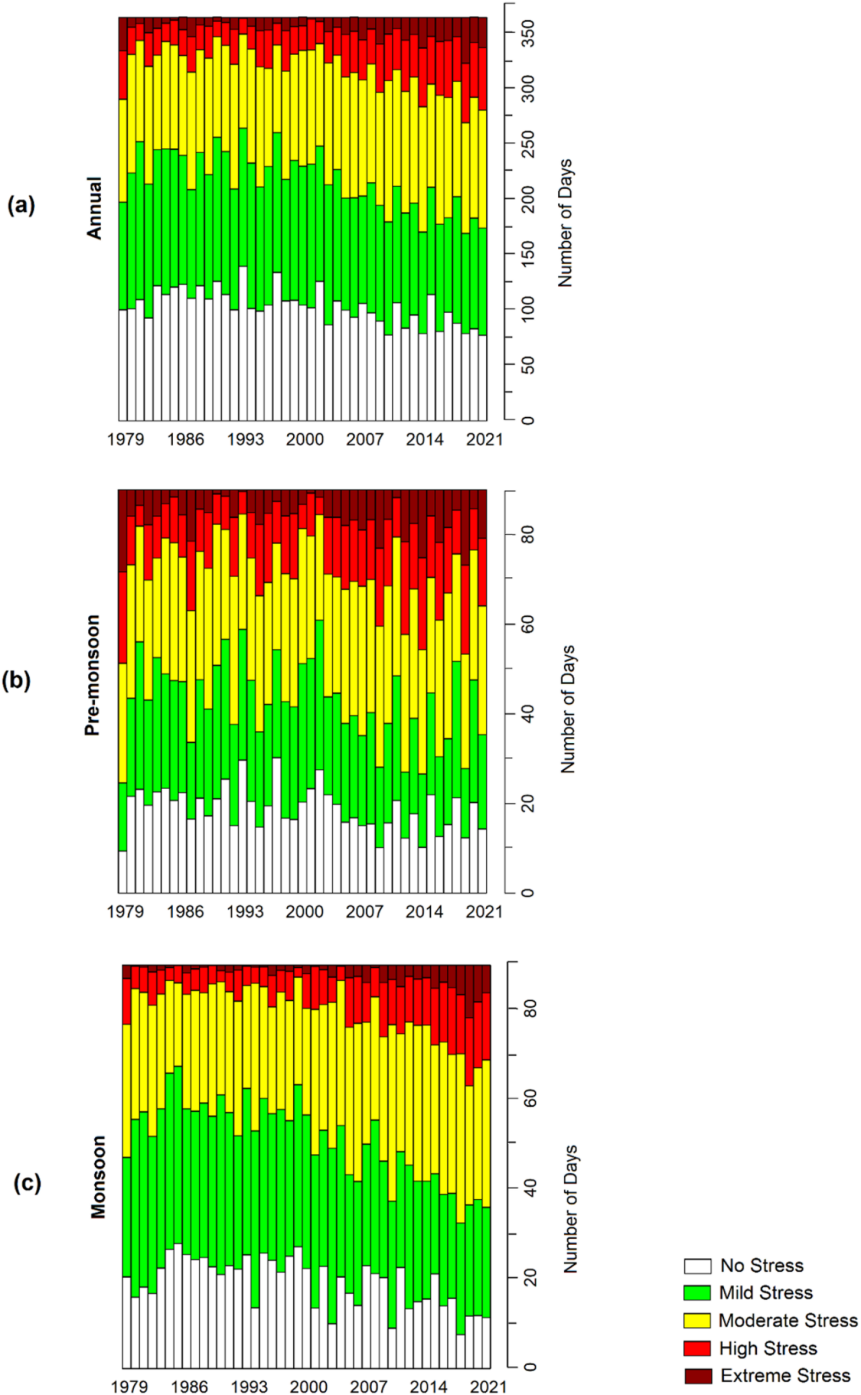
The number of affected days by different categories of average heat stress during (**a**) annual, (**b**) pre-monsoon, and (**b**) monsoon from 1979 to 2021 in Bangladesh.

Figure 7 illustrates the number of days falling into different categories of WBGT throughout the study period. The areal average daily maximum WBGT was used to estimate these categories for the entire country. The results indicate that as WBGT increases, both the affected area and duration also increase. There is a rise in high and extreme heat stress periods on an annual and seasonal scale, accompanied by a decrease in periods of no heat stress or moderate heat stress. Before 2001, the number of extreme heat stress days annually was less than five, but in recent years, it has exceeded ten days. Similarly, the number of high-heat stress days was less than 25 in the early years but has reached nearly 50 days in recent years. Similar trends in high and extreme heat stress days were observed for both the pre-monsoon and monsoon seasons, with the highest increase seen during the monsoon. The number of days of high and extreme heat stress during the monsoon has tripled in recent years compared to the early period. Conversely, there has been a significant decrease in the number of no and mild heat stress days during the monsoon.

### Trends in the drivers of WBGT

Analyzing the trends of the drivers of WBGT, including Ta, specific humidity (SH), WS, and SR, is important to understand the factors contributing to the increasing WBGT in Bangladesh. Considering RH's relation with Ta, SH instead of RH was used to assess the actual impact of moisture on the WBGT. Therefore, this section examines the trends of these drivers. Supplementary Figure S3 presents the geographical variability of the yearly and seasonal changes in the mean and maximum daily SR. The results show a decrease in SR in the western part of the country. The annual average SR has decreased by 2–4 Wm^−2^/decade in the west, while there are some points in the far west where pre-monsoon SR has increased by 2–6 Wm^−2^/decade from 1979 to 2021. The trends in the annual and seasonal average of daily maximum SR also reveal a decrease in the west, with a decline of 2–8 Wm^−2^/decade, much higher than the average SR. The decrease in SR is more pronounced for annual and pre-monsoon periods than the monsoon.

Supplementary Figure S4 displays the geographical distribution of the changes in SH. The results show an increase in specific humidity over almost the whole of Bangladesh, both annually and seasonally. The annual specific humidity increased in the range of 0.3 to 0.7 g kg^−1^/decade, with the highest increase in the north and the lowest in the southeast. A similar pattern was observed for pre-monsoon and monsoon seasons. However, the increase was higher for the pre-monsoon, ranging from 0.4 to 0.9 g kg^−1^/decade. The southeast region showed no change in specific humidity during the pre-monsoon season and a slight increase in the monsoon by 0.2 to 0.3 g kg^−1^/decade. The annual and pre-monsoon maximum specific humidity also increased over most of the country, except for the northwest. The monsoon maximum specific humidity increased across the whole country.

The trend analysis of Ta reveals a rise in the average annual and monsoon Ta across Bangladesh, with an average increase of 0.5 to 1.0 °C in the pre-monsoon season in the eastern part of the country (Supplementary Figure S5). The yearly and seasonal daily maximum Ta increased in Bangladesh's east and central regions, ranging from 0.5 to 2 °C. The northeastern hilly region experiences even higher increases.

Trends in WS show minimal changes in average and maximum values for annual and seasonal scales (Supplementary Figure S6). The yearly and monsoon maximum WS only increased in the central region by 0.2 to 0.3 m s^−1^/decade. At the same time, the pre-monsoon average WS decreased in the southern part of the central region, and the maximum WS decreased over a small area in the northeast.

The spatial analysis of the maps revealed that the most influential factor in increasing WBGT in the country is the rising Ta. The higher annual and seasonal WBGT increase in the east is primarily attributed to a faster rise in Ta in that region. The southeast region experienced the highest rise in annual WBGT due to the significant increase in air temperature. The SH has also increased over most of the country, like WBGT, indicating its high influence on WBGT. Its influence is particularly noticeable in the central region where WBGT increased even though Ta did not increase significantly. However, the increase in average annual and monsoon SH in the northwest region was insufficient to raise the WBGT temperature significantly in that area. Although the annual average temperature increased throughout Bangladesh, the increase in mean WBGT was lower in the western part of the country, which could be linked to the declining SR in that region. Overall, annual and seasonal SR decreased in the west, coinciding with a lesser increase in WBGT. However, the decreased SR had a relatively smaller impact in the west than in the east.

In contrast, the influences of WS were relatively minor. The increased annual and monsoon maximum WS in the central region was inadequate to counterbalance the rising WBGT. Overall, the results suggest that the primary factor contributing to the elevated WBGT temperature in the country is the increase in air temperature, followed by SH.

**Figure 8:**
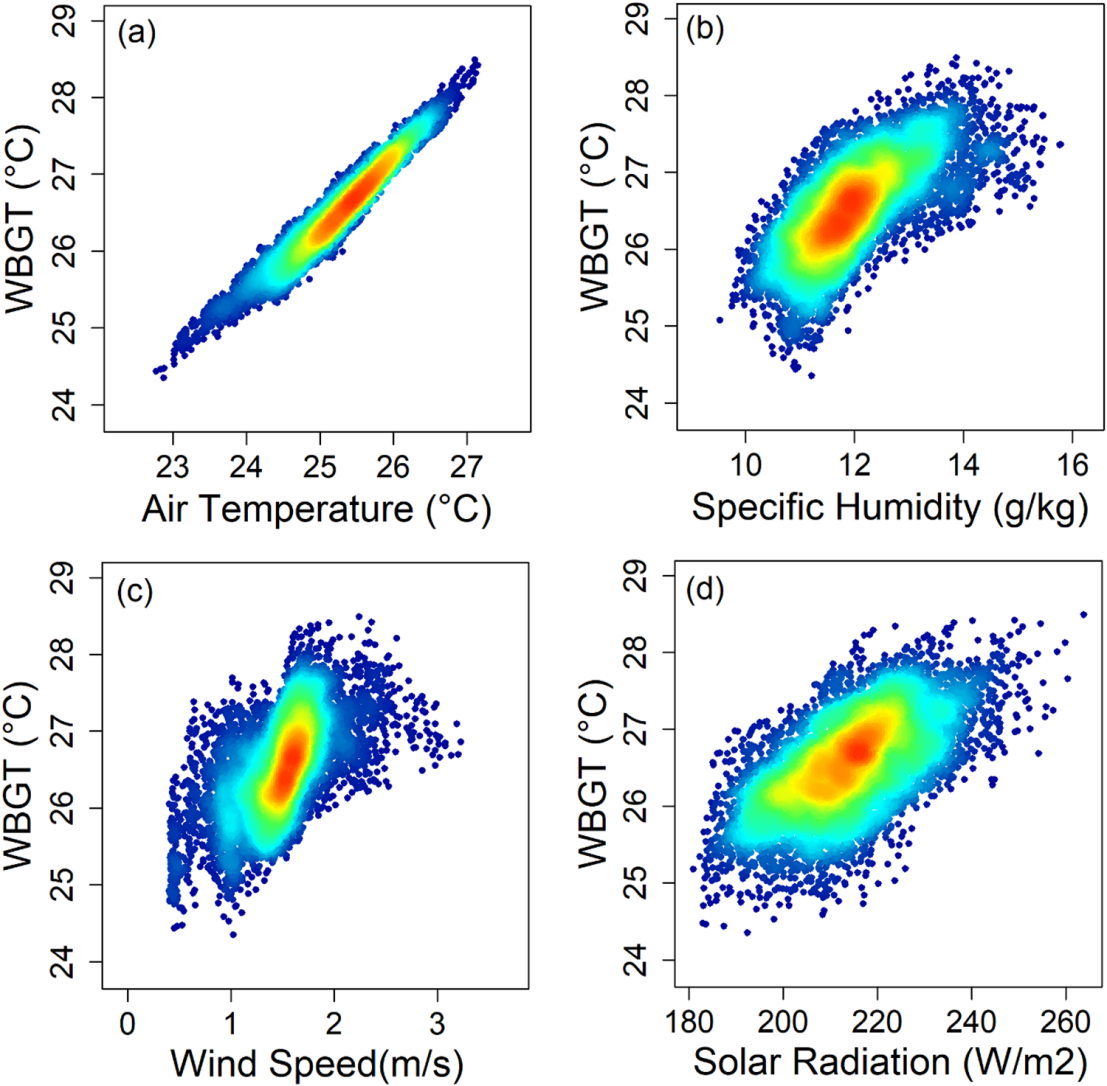
Scatter plots showing the relationship between WBGT and (**a**) solar radiation, (**b**) relative humidity, (**c**) 2 m temperature, and (**d**) wind speed.

Figure 8 presents density scatter plots illustrating the influence of the drivers on WBGT. The plots use red to represent areas with high data density and blue for lower-density regions. The high-density patch on the plot confirms the expected positive association of SR, RH, and Ta with WBGT. The nonparametric Kendall-tau revealed a significant association (*p* < 0.01) of WBGT with a correlation coefficient of 0.87 for Ta, 0.52 for SH, 0.43 for SR and 0.45 for WS. A partial correlation analysis was conducted to isolate the unique relationship between WBGT and each driver by removing the effects of other drivers. The results revealed partial correlations of 0.99, 0.92, 0.87 and − 0.89 for Ta, SH, SR, and WS with WBGT, all significant at *p* < 0.01. These findings indicate that Ta most strongly influences WBGT, followed by SH, WS, and SR.

An MLR model was also utilized to assess the relative importance of the drivers. The dataset was standardized to ensure comparability, and the MLR model was fitted by regressing the response variable on the predictor variables. The standardized coefficients obtained from the model were used to calculate the absolute values, indicating the magnitude of each variable's impact on the response. These importance values were scaled between 0 and 1, enabling a fair comparison across drivers. The scaled relative importance values were then presented in a bar plot (Supplementary Figure S7 online), offering a concise representation of the contributions of each driver to WBGT. The result was very similar to that obtained using partial correlation. The importance values indicated Ta as the primary driver behind Bangladesh's rising WBGT. It influences WBGT by 85.9%, followed by SH (15.4%), WS (-4.2%) and SR (2.9%).

## Discussion

This study analyzed the spatiotemporal changes in outdoor WBGT across Bangladesh from 1979 to 2021 using Liljegren's model. Simplified WBGT (sWBGT) estimation methods are often used to avoid the complexities of the Liljegren method. However, Kong and Huber^11^ compared the Liljegren method with an sWBGT method^75^ and found that the simplified method significantly overestimates heat stress in hot and humid areas. They also demonstrated systematic overestimation of labor loss using sWBGT worldwide and recommended discontinuing its use. Recently, Brimicombe et al.^76^ developed a new sWBGT method that showed similar efficiency to the Liljegren method in estimating WBGT. However, while this new method accurately captured the geographical variability of WBGT, it underestimated extreme WBGT values. Brimicombe et al.^76^ also reported WBGT anomalies of ± 2°C using this simplified method. Therefore, this study avoids using simplified methods to provide a reliable estimate of WBGT for Bangladesh.

The spatial distribution of annual and seasonal daily average and maximum WBGT in Bangladesh, as depicted in Fig. 1, reveals higher average and maximum WBGT in the northwest and lower in the southeast, annually and in different seasons. The hilly areas of the southeastern region exhibit the lowest WBGT values (Supplementary Figure S1). During the pre-monsoon season, WBGT values are generally higher than during the monsoon season across the country. Upon comparing WBGT values with the Köppen-Geiger climate classification^45,46^, it becomes evident that WBGT values are higher in the temperate, dry winter, hot summer (Cwa), and tropical savannah (Aw) zones while lower in the tropical monsoon (Am) zone.

Figure 2 illustrates the nationwide increase in WBGT at varying rates. Higher rates of change are observed in the southwestern and northeastern regions, located within the tropical monsoon zone of the Köppen-Geiger climate classification. Conversely, lower change rates are observed in the central, western, and northwestern regions, primarily in temperate, dry winter, hot summer, and tropical savannah zones. During the pre-monsoon season, the southeastern areas exhibit higher change rates for the maximum WBGT than the monsoon season. In contrast, the change rates are higher in other areas during the monsoon season. Similar patterns can be observed for average changes, with higher change rates in the southeastern and northeastern areas during the pre-monsoon season compared to the monsoon season.

Figures 6 and 7 demonstrate an increasing trend in the number of days and affected areas with higher WBGT values (moderate, high, and stress conditions) while decreasing for categories with lower WBGT values. Further insights are provided in Figs. 3, 4, and 5. In some locations, the number of days and affected areas for moderate, high, and stress conditions have decreased, which does not necessarily indicate a reduction in WBGT values. Rather, in most cases, the decline is attributed to an increase in other higher category WBGT values or an increase in WBGT during another season in the same location. For example, Figs. 3 and 5 display the annual and pre-monsoon average changes in the number of days per decade. Moderate stress conditions have decreased in many locations, while high-risk conditions have increased. The number of days per decade with extreme conditions during the pre-monsoon season has decreased in the northwest and increased in the central and northwestern areas (Fig. 4). Conversely, the number of days per decade with extreme conditions during the monsoon season in those areas has increased (Fig. 5). However, Fig. 3 indicates a decrease in the annual average extreme stress conditions in some southeastern locations but an increase in moderate and high-stress conditions with higher rates, indicating an overall increase in WBGT.

Comparing the spatial changes in the drivers (Supplementary Figure S3-S6) with the changes in WBGT (Fig. 2) reveals a stronger spatial correlation between SR and Ta with the changes in WBGT. The rate of WBGT increase was higher in the southeast, where the rate of SR decrease was lower, and the rate of Ta increase was higher, and vice versa in the northwest. RH did not show any significant change, indicating no impact on the changes in WBGT. Similarly, WS did not exhibit a strong spatial correlation with WBGT changes. This inadequate response of RH and WS compared to the other variables is one of the limitations of WBGT measurements^7^. This observation is also evident in the scatter plots of each driver with WBGT (Fig. 6) and in the bar chart showing the relative importance of each driver in the MLR model of WBGT (Supplementary Figure S7). These findings clearly suggest that Ta strongly influences WBGT, followed by SH, WS and SR.

Previous studies evaluating WBGT in Dhaka, Bangladesh, were limited to a single study utilizing daily data from April to September 2016^38^. Research related to WBGT in South Asian countries is also scarce^36,37,77^. The study by Jacobs et al.^38^ demonstrated that WBGT in Dhaka reached almost 35°C during the daytime, even when air temperature decreased after the onset of the monsoon, which supports the present study's findings. The spatial distribution of daily maximum WBGT over Bangladesh (Fig. 3) illustrates that annual and pre-monsoon WBGT can reach 34°C in the western part of the country. WBGT trends indicate a rise of 0.1 to 0.5°C per decade across Bangladesh (Fig. 4), consistent with the estimate of Ullah et al.^77^ for South Asia, which reported a WBGT change of 6.5°C over a century. The present study highlights a significant increase in the annual and seasonal frequency of extreme WBGT days (Fig. 5). Li^78^ also demonstrated that Southeast Asia, bordering southeast Bangladesh, is experiencing more frequent, longer-lasting, and intense heatwaves.

Only one study in South Asia attempted to identify the cause of increasing WBGT temperatures^36^. They revealed that moist air from the Arabian Sea is the primary driver of extreme WBGT in Southern Pakistan. Studies in other regions have also emphasized the significant effect of humidity on WBGT^78,79^. The WBGT estimation method assigns more weight to the natural wet bulb temperature (70%) than air temperature (10%). Therefore, the influence of RH on WBGT is naturally higher than that of air temperature. Though SH showed an increase in most of Bangladesh, RH may not have changed significantly due to the increase in temperature. Vicente-Serrano et al.^80^ analyzed global changes in RH from 1979 to 2014 and found a predominantly negative trend at the global scale, primarily attributed to the rise in temperature, which increased the moisture-holding capacity. Therefore, the present study indicates that rising temperatures mainly drive the increasing trends in WBGT over Bangladesh.

The rising WGBT in Bangladesh poses imminent risks to human health and well-being. With increasing WBGT levels, the population faces heightened threats of heat-related illnesses, placing vulnerable groups at particular risk. Heatwaves can lead to dehydration, heat exhaustion, and heatstroke, exacerbating health disparities and straining healthcare resources. Furthermore, elevated WBGT levels can impair cognitive function, labor productivity, and overall quality of life, impacting individuals' physical and mental well-being. Urgent action is required to implement heat adaptation measures, enhance access to cooling facilities, and raise awareness about heat-related risks to mitigate the adverse health impacts of rising WBGT.

This research offers a pioneering exploration into the dynamics of WBGT by utilizing high-resolution gridded reanalysis climate data. By comprehensively analyzing spatiotemporal shifts in WBGT, this study fills a critical knowledge gap in understanding climate-related changes within Bangladesh. The outcomes of this investigation hold significant potential for informing strategic adaptations and effective measures to counter the influence of climate change on health outcomes and mortality rates. This is particularly pertinent given the susceptibility of economically vulnerable populations, especially those involved in construction and agriculture, to the adverse impacts of excessive heat^42^. Consequently, this research has the potential to facilitate targeted interventions and policies aimed at safeguarding public health and well-being in the face of changing climate conditions.

## Conclusion

In this study, WBGT values were calculated using several climatic variables from ERA5 datasets, and their spatiotemporal variations were assessed based on trend analysis using the M–K test and Sen's slope estimator for Bangladesh. The WBGT values were categorized into five categories to examine their spatiotemporal variations. The main conclusions of this study can be summarized as follows:

1. The average annual WBGT in the country varies between 25 °C in the far southeast and 31 °C in the northwest, with maximum values exceeding 33 °C in the western half of the country, reaching a maximum of 34.6 °C in the northwest.
2. WBGT values are increasing across the country at rates ranging from 0.08 to 0.5 °C per decade. The increases were higher in the southeast and northeast, where WBGT is relatively lower and lower in the west, where WBGT is higher. The highest increase was observed in the annual average and pre-monsoon maximum WBGT in the southeast, with a rate of over 0.5 °C per decade.
3. The number of days with WBGT corresponding to high and extreme risk of heat-related illness has increased by 2–4 days per decade in the monsoon season and 1–3 days per decade in the pre-monsoon season from 1979–2021. The results indicate that the increase in WBGT has led to a five-fold increase in affected areas and a three-fold increase in days of high and extreme heat stress during the monsoon season in recent years compared to the earlier period.
4. The primary contributor to the increasing WBGT and associated health risks in Bangladesh, as determined by trend and relative importance analyses of various climatic drivers, is air temperature, followed by solar radiation, wind speed, and relative humidity.

The spatiotemporal variability of WBGT, which provides a more accurate measure of the impact of heat stress on human health, has not been previously studied over the entire extent of Bangladesh for such an extended period. Approximately 37.75% of Bangladesh's population of 160 million is engaged in monsoon agriculture, experiencing prolonged exposure to heat stress. High temperatures have been associated with increased mortality in Bangladesh, with the elderly, children, men, and urban populations particularly vulnerable. Additionally, 5.3 million urban residents rely on outdoor labor for their livelihoods. The increasing WBGT poses a significant heat risk to a large population, particularly in terms of occupational injuries, heat-related illnesses, and deaths. To mitigate the escalating effects, the country needs region-specific guidelines for activity adjustments, such as work-rest ratios, activity duration and timing, hydration, and appropriate attire. The data from this study can be used to prepare for adaptation, reform policies, and mitigate the impacts of climate change-related diseases and deaths, especially considering the large number of economically vulnerable individuals working in agriculture and construction who are exposed to extreme heat in the country.

While this study offers valuable insights into WBGT trends and their health implications in Bangladesh, it's important to acknowledge its limitations. Reliance on reanalysis datasets may introduce uncertainties despite validation efforts by other researchers such as Yildiz et al.^51^. Additionally, the focus solely on climatic variables overlooks other potential influences. Future studies could address these limitations by incorporating more ground-based validation using observations and conducting longitudinal analyses. Collaborative efforts between researchers and relevant stakeholders can enhance data collection and analysis, providing more reliable insights for effective strategies to mitigate the adverse health effects of rising WBGT.

### Supplementary Information

Supplementary Figures.

## Data Availability

Data used in this study are available in the public domain (https://cds.climate.copernicus. eu/cdsapp#!/dataset/reanalysis-era5-singlelevels?tab = overview). Also, the datasets used and/or analyzed during the current study are available from the corresponding author upon reasonable request.

## Code availability

The codes used for data processing can be provided on request to the corresponding author.
