# Supplementary material for: Changes in wet bulb globe temperature and risk to heat-related hazards in Bangladesh
Source: Sci Rep. 2024 May 6;14:10417. doi: 10.1038/s41598-024-61138-8 (PMC11074116; doi:10.1038/s41598-024-61138-8)
Supplement: Supplementary file 1 — Supplementary Figures. [file 41598_2024_61138_MOESM1_ESM.docx]

**Changes in Wet Bulb Globe Temperature and Risk to Heat-Related Hazards in Bangladesh**

A S M Maksud Kamal^1*^, Abul Kashem Faruki Fahim^1^, and Shamsuddin Shahid^2*^

^1^Department of Disaster Science and Climate Resilience, University of Dhaka, Dhaka-1000, Bangladesh (email: [maksudkamal@du.ac.bd](mailto:maksudkamal@du.ac.bd); [farukifahim@.du.ac.bd](mailto:farukifahim@.du.ac.bd))

^2^Department of Water & Environmental Engineering, School of Civil Engineering, Faculty of Engineering, Universiti Teknologi Malaysia, Johor Bahru 81310, Malaysia (email: [sshahid@utm.my](mailto:sshahid@utm.my))

*Corresponding author. Email: [maksudkamal@du.ac.bd](mailto:maksudkamal@du.ac.bd); sshahid@utm.my


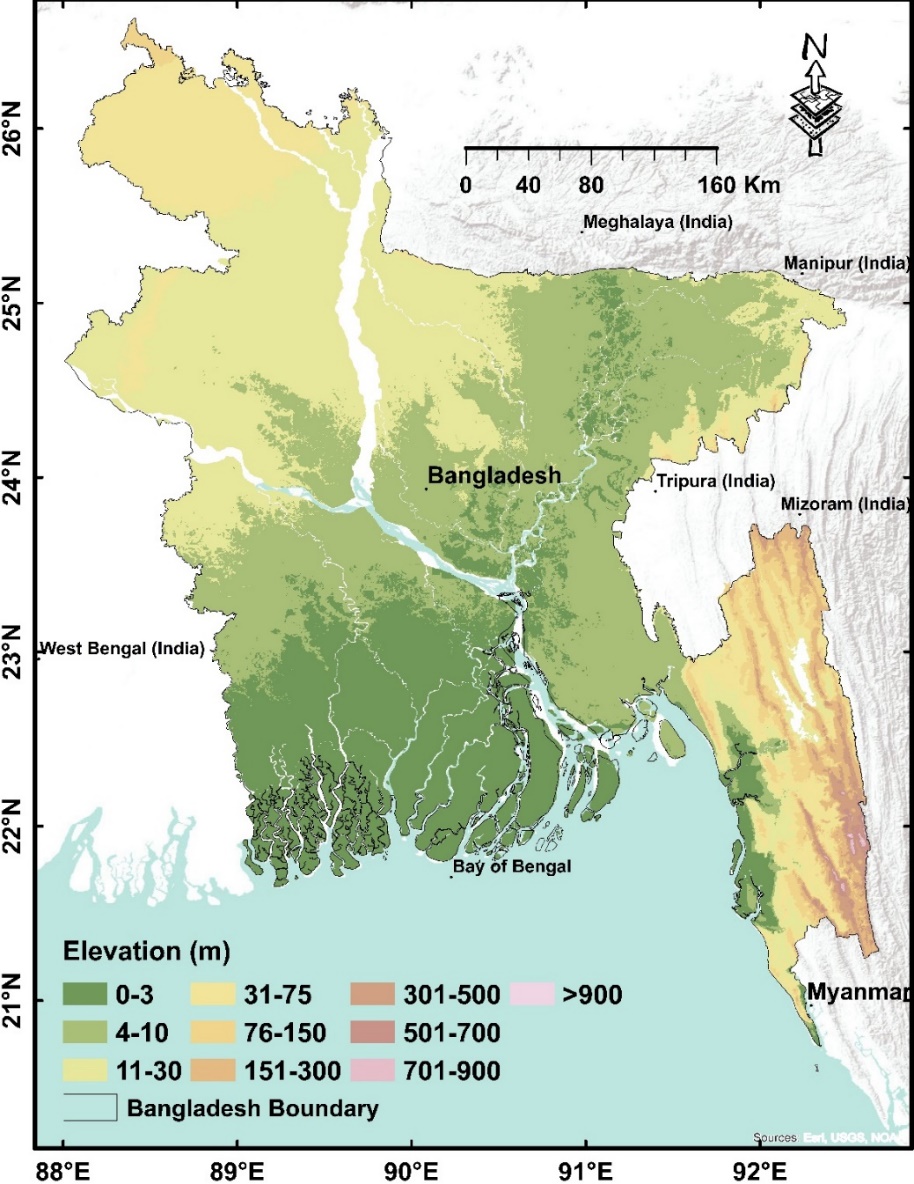


**Supplementary Figure S1.** Topography of Bangladesh (after Fahim et al. ^41)^.


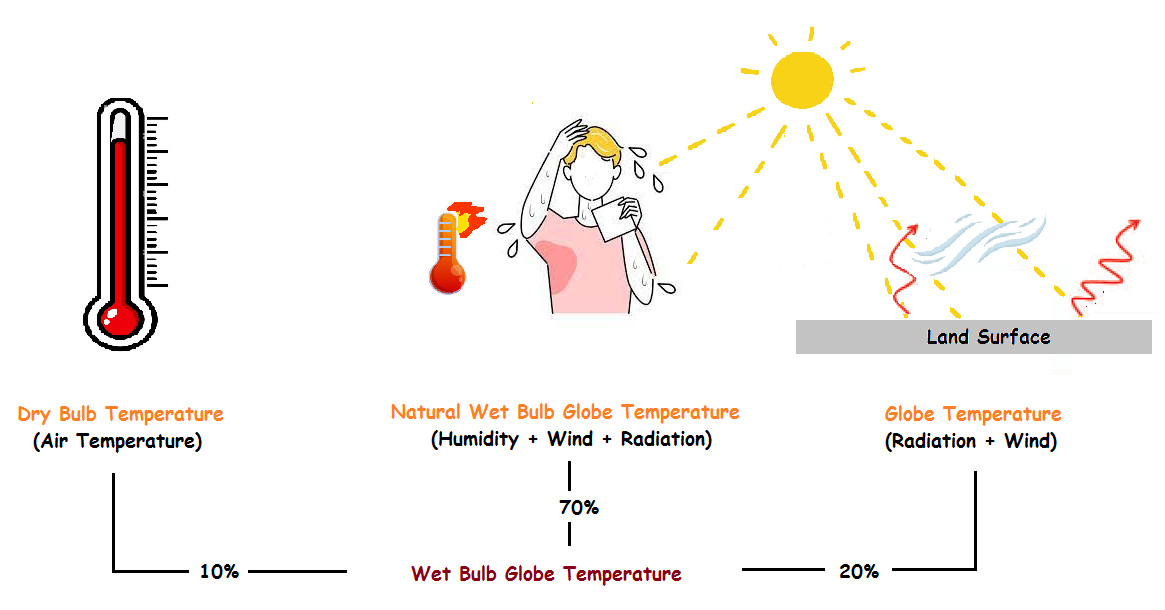


**Supplementary Figure S2.** Estimation of wet bulb globe temperature from atmospheric and psychrometric components


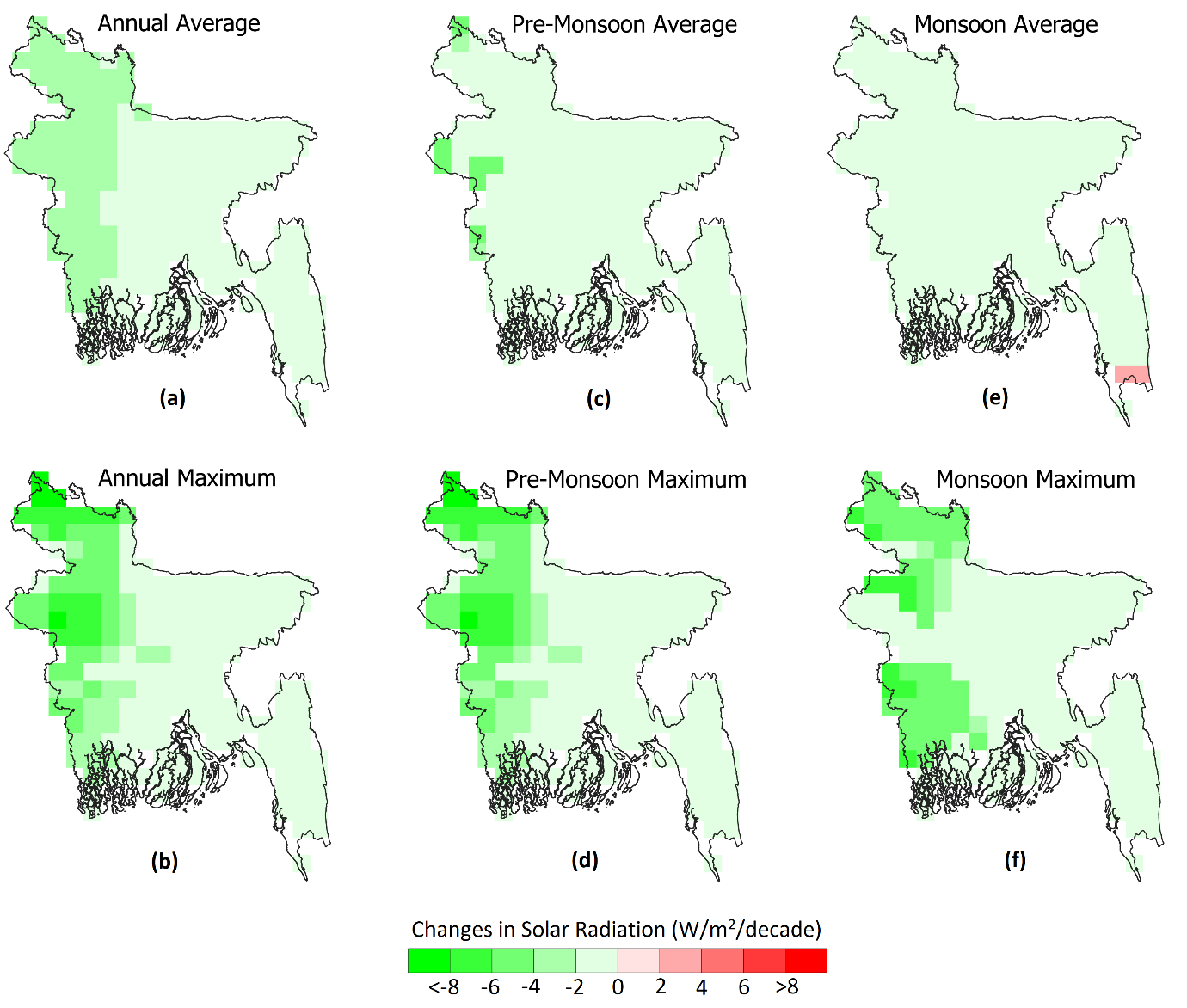


**Supplementary Figure S3.** The spatial variability of the rate of change (Wm^-2^/decade) in (a) annual maximum, (b) pre-monsoon maximum, (c) monsoon maximum, (d) annual average, (e) pre-monsoon average, and (f) monsoon average of solar radiation ((Wm^-2^) calculated using Sen's slope estimator. Only significant changes estimated using the MK test are presented in the figure.


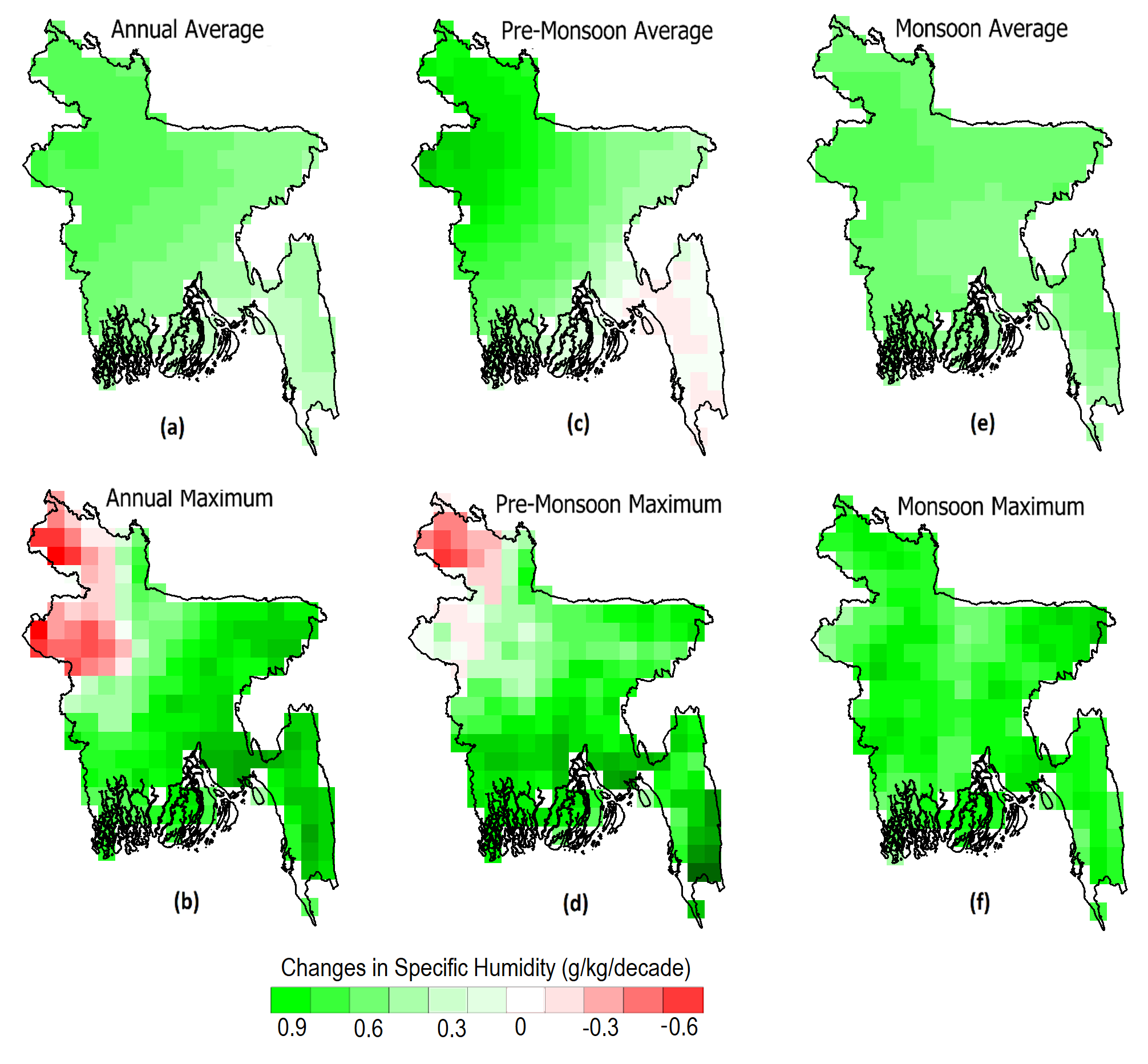


**Supplementary Figure S4.** The spatial variability of the changes (g/kg/decade) in (a) annual maximum, (b) pre-monsoon maximum, (c) monsoon maximum, (d) annual average, (e) pre-monsoon average, and (f) monsoon average of specific humidity (g/kg) calculated using Sen's slope estimator. Only significant changes obtained by the MK test are presented in the figure.


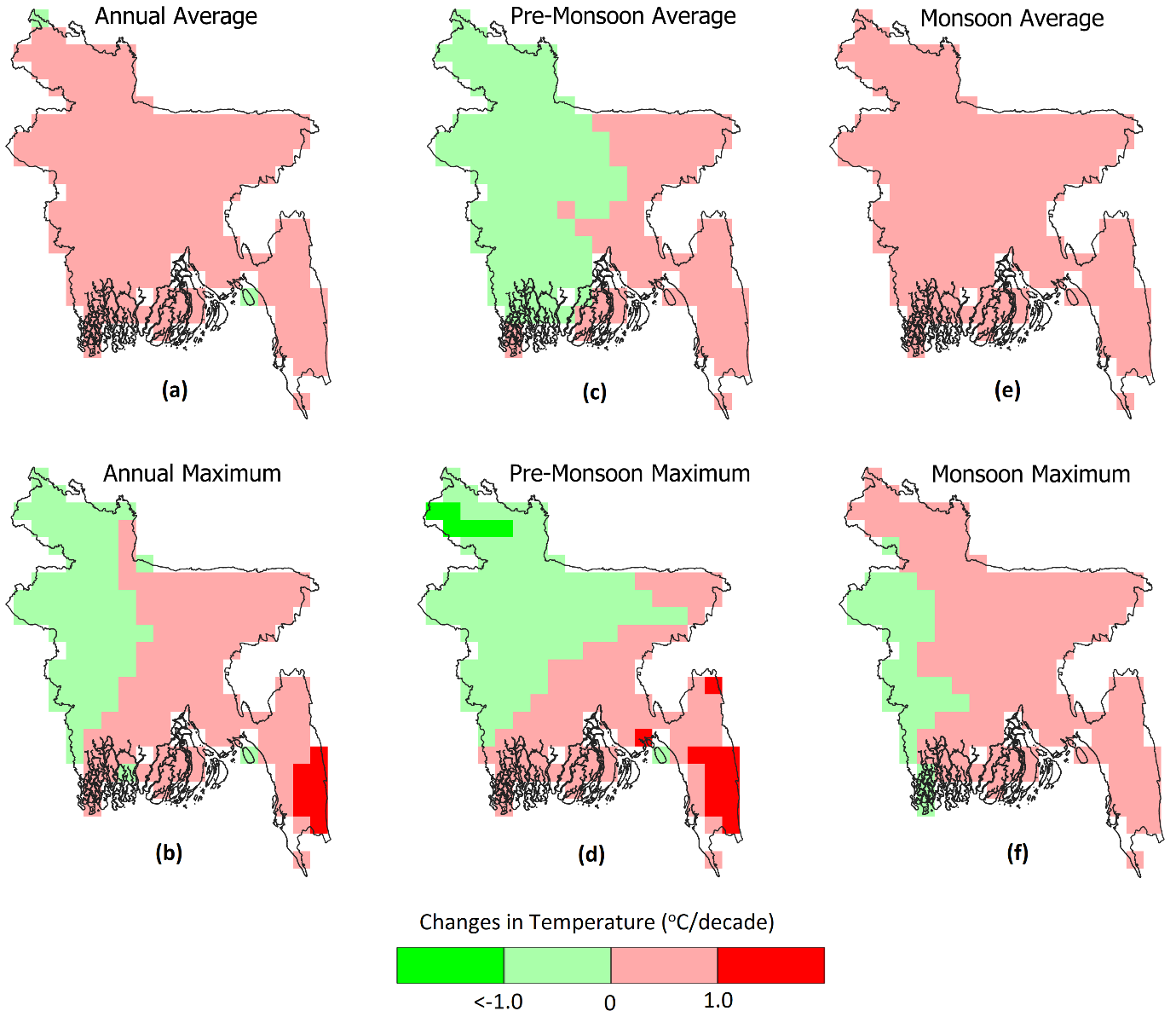


**Supplementary Figure S5.** The geographical distribution of the changes (ºC/decade) in (a) annual maximum, (b) pre-monsoon maximum, (c) monsoon maximum, (d) annual average, (e) pre-monsoon average, and (f) monsoon average of 2m temperature (ºC) calculated using Sen's slope estimator. Only significant changes estimated using the MK test are shown in the figure.


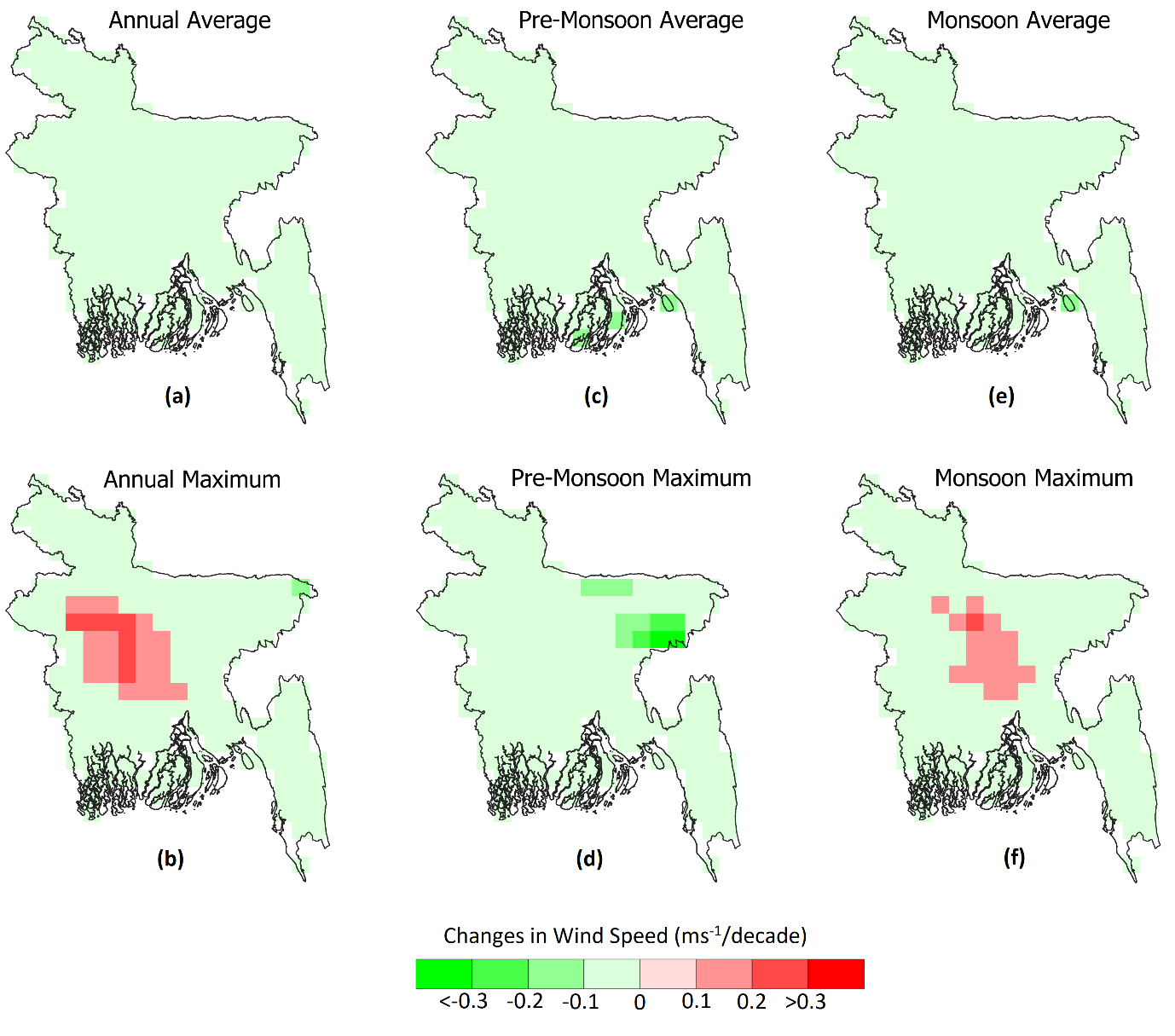


**Supplementary Figure S6.** The geographical distribution of the changes (m/s/decade) in (a) annual maximum, (b) pre-monsoon maximum, (c) monsoon maximum, (d) annual average, (e) pre-monsoon average, and (f) monsoon average of wind speed (m/s) calculated using Sen's slope estimator. Only significant changes estimated using the MK test are presented in the figure.


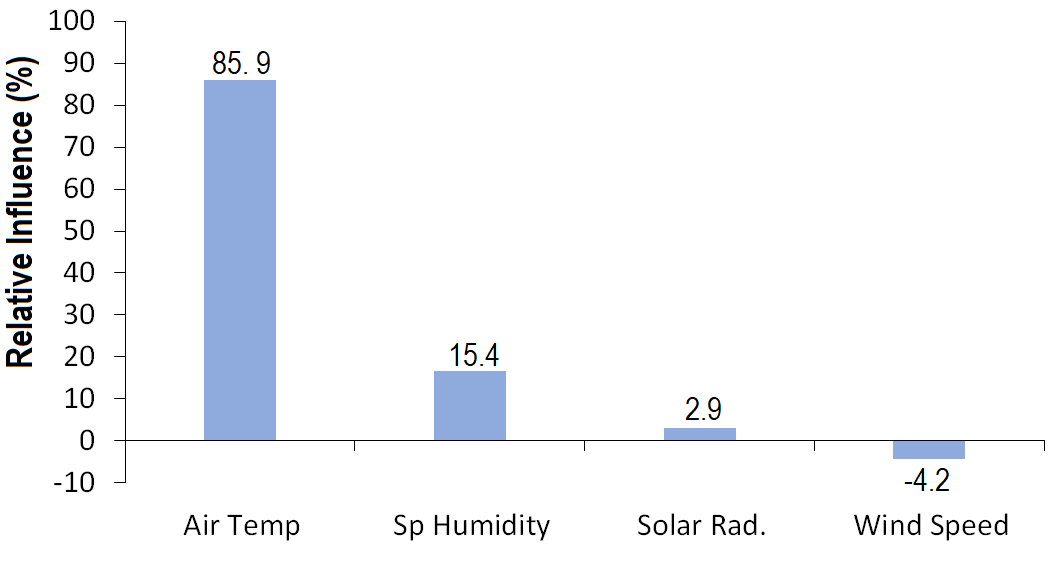


**Supplementary Figure S7.** The relative fluence of drivers on WBGT in MLR.
